# COVID-19 Heart Lesions in Children: Clinical, Diagnostic and Immunological Changes

**DOI:** 10.3390/ijms24021147

**Published:** 2023-01-06

**Authors:** Elena Vasichkina, Daria Alekseeva, Igor Kudryavtsev, Anzhela Glushkova, Anastasia Y. Starshinova, Anna Malkova, Dmitry Kudlay, Anna Starshinova

**Affiliations:** 1 Almazov National Medical Research Centre, 197341 St. Petersburg, Russia; 2 H. Turner National Medical Research Center for Children’s Orthopedics and Trauma Surgery of the Ministry of Health of the Russian Federation, 196603 St. Petersburg, Russia; 3 Institute of Experimental Medicine, 197341 St. Petersburg, Russia; 4 V.M. Bekhterev National Research Medical Center for Psychiatry and Neurology, 192019 St. Petersburg, Russia; 5 Medical Department, Saint Petersburg State Pediatric Medical University, 194100 St. Petersburg, Russia; 6 Faculty of Natural Sciences, Ariel University, Ariel 40700, Israel; 7 Pharmacology Department, I.M. Sechenov First Moscow State Medical University, 119435 Moscow, Russia; 8 NRC Institute of Immunology FMBA of Russia, 115478 Moscow, Russia

**Keywords:** myocarditis, children, coronavirus infection, COVID-19, SARS-CoV-2, Post-COVID-19, cardiovascular inflammation, T cells, pathogenesis

## Abstract

In the beginning of COVID-19, the proportion of confirmed cases in the pediatric population was relatively small and there was an opinion that children often had a mild or asymptomatic course of infection. Our understanding of the immune response, diagnosis and treatment of COVID-19 is highly oriented towards the adult population. At the same time, despite the fact that COVID-19 in children usually occurs in a mild form, there is an incomplete understanding of the course as an acute infection and its subsequent manifestations such as Long-COVID-19 or Post-COVID-19, PASC in the pediatric population, correlations with comorbidities and immunological changes. In mild COVID-19 in childhood, some authors explain the absence of population decreasing T and B lymphocytes. Regardless of the patient’s condition, they can have the second phase, related to the exacerbation of inflammation in the heart tissue even if the viral infection was completely eliminated—post infectious myocarditis. Mechanism of myocardial dysfunction development in MIS-C are not fully understood. It is known that various immunocompetent cells, including both resident inflammatory cells of peripheral tissues (for example macrophages, dendritic cells, resident memory T-lymphocytes and so on) and also circulating in the peripheral blood immune cells play an important role in the immunopathogenesis of myocarditis. It is expected that hyperproduction of interferons and the enhanced cytokine response of T cells 1 and 2 types contribute to dysfunction of the myocardium. However, the role of Th1 in the pathogenesis of myocarditis remains highly controversial. At the same time, the clinical manifestations and mechanisms of damage, including the heart, both against the background and after COVID-19, in children differ from adults. Further studies are needed to evaluate whether transient or persistent cardiac complications are associated with long-term adverse cardiac events.

## 1. Introduction

The World Health Organization (WHO) in March 2020 declared COVID-19 a pandemic, pointing to rapid global spread of SARS-CoV-2, the virus that causes COVID-19 [1]. The novel coronavirus infection (COVID-19) turned the world upside down because it is a serious global threat both for population health on the one hand, and it necessitated the study of new pathological conditions and immunologic changes associated with the infection development and comorbidity and its impact on patients with COVID-19, on the other hand. Additionally, it triggered the need to find new methods of diagnosis and treatment not only for this infection, but also for the consequences of COVID-19 [2,3].

As of October 2022, 617 million confirmed cases of COVID-19 and over 6.5 million deaths were reported globally. The most confirmed cases of coronavirus (COVID-19) were across the whole of Europe, with over 255 million cases, followed by the United States of America—over 178 million cases. The Russian Federation had over 21 million cases. Pathogenesis of myocardial damage against the background of COVID-19 (https://www.who.int/publications/m/item/weekly-epidemiological-update-on-COVID-19---5-october-2022, accessed on 5 October 2022).

## 2. Characteristics of SARS-CoV-2

Currently, it is known that SARS-CoV-2 is a variant of bate coronavirus that belongs to the family of *Coronaviridae*. It is spherical and pleomorphic, and measures between 70 and 110 nm in length, containing single-stranded (positive-sense) RNA and dotted with spike glycoproteins [4,5].

The genome size of the SARC-CoV-2 is about 30 kb. It comprises genes S, M, E, and N, located in 10 and 11 open reading frames, encoding structural proteins including surface (S), envelope (E), membrane (M), and nucleocapsid (N) proteins. All these proteins are responsible for the replication and the spread of the virus. (S) protein visually forms a characteristic crown shape in a viral envelope, thanks to it, the virus got its name. (S) protein, in turn, consists of the S1 and S2 subunits. S1 subunit contains a signal peptide, followed by an N-terminal domain (NTD) and receptor-binding domain (RBD). S2 includes fusion peptides inner membranes, two sequences of heptapeptide repeats, the membrane proximal external region and the transmembrane domain. Exactly, the S2 threat helps the genome integrate into the host by fusion its membranes and the virus’s membrane [6].

The trimeric spike (S) protein of SARS-CoV-2 is a basic surface glycoprotein that connects to ACE2 (*Angiotensin-converting enzyme* 2) to enter the host cell using serine proteases 2 cells for priming S-protein. S-protein is the most immunogenic component of the pathogen and therefore, the most powerful target for for neutralizing antibodies and inhibiting viral infection. M-protein is the most abundant structural protein that determines the form of the virion and plays a predominant role in the process of budding viral particles from their hosts’ cells. E-protein is necessary for viral infection and replication [7]. Assembling of S, E, M creates a viral envelope. T-protein connects with genomic RNA and maintains genetic material in a viral envelope. It is an important protein for replication of the virus. During self-assembly of the virus particles, the M-protein interacts with other structural proteins, forming a complete virion [6,8].

It is known, all viruses evolve as time passes. SARS-CoV-2 is not exception [9]. COVID-19 was reported as a new type of upper-respiratory tract disease among chickens in 1937 and in 1965 D. Tyrrell and M. Bynoe cultured human coronavirus from an embryonic epithelium of the nasopharyngeal mucosa [10]. There used to be four coronaviruses in the human population (HCoV-229E, -OC43, -NL63, and -HKU1), which enter the host cell and bind to a beta-alanine receptor [11].

Today, thanks to numerous studies, WHO declared several genome variants of SARS-CoV-2 (Alpha, Beta, Delta, Gamma, Epsilon, Eta, Iota, Kappa, N/A, Zeta, Mu), performing their characteristics regarding the transmissibility, the virulence, the severity of the disease and possible adverse effects of vaccination [12]. In particular, until recently, WHO and the Centers for Disease Control and Prevention variants Alpha, Beta, Delta, and Gamma were categorized into a virus group, provoking anxiety [13]. According to the WHO data, as of 31 August 2021—Alpha, Beta, Delta, and Gamma variants spread to 193, 141, 91 and 170 countries, respectively [14,15].

The emergence of new variants of SARS-CoV-2 B.1.1.529 Omicron caused a new wave of concern. The strain was detected in South Africa for the first time (https://www.who.int/activities/tracking-SARS-CoV-2-variants, accessed on 5 October 2022). Omicron is currently the dominant variant. It has the high transmissibility and moreover, the ability to escape from immune response, caused by vaccination or natural infection [16]. In addition, its pathogenicity is much lower and it causes less hospitalization, lethal outcomes and severe cases [17,18]. However, it should be noted, in comparison with Delta variant, Omicron increased a number of hospitalizations among children aged 5–11 [19,20].

## 3. Clinical Symptoms of COVID-19 in Children

It is clear today that the severity of the clinical course of COVID-19 depends on strains of SAR-CoV-2 and the presence of concomitant pathology, age, race, gender, genetic factors, and characteristics of the immune response as well [20,21]. Recently, there were reports about genetic factors that affect susceptibility and the severe illness of COVID-19. Several genome-wide association studies, focusing on the contribution of common genetic variations 12–15 locus during COVID-19 proved connection with several genomic locus, particularly 3p21.31 locus 12–16, with the severity of SARS-CoV-2 and susceptibility to infection [22].

In the beginning of COVID-19, the proportion of confirmed cases in the pediatric population was relatively small and there was an opinion that children had often the mild or asymptomatic course of infection [23,24,25,26]. According to the literature, incidence of novel coronavirus infection in the pediatric population is relatively low compared to adults and ranges from 1% to 16% [27,28,29]. In most children with COVID-19, the disease is mild, moreover, asymptomatic infection is registered in 15–42% of children [30]. Mild COVID-19 in childhood lead some authors to explain the absence of population decreasing T and B lymphocytes [27,31]. Other authors have suggested a protective role prevention of tuberculosis [32]. Some studies suggest a theory of mild COVID-19 in children, caused by difference in the expression of the ACE2 receptor that is necessary for attaching SARS-CoV-2 [33,34,35]. Moreover, difference in the clinical picture of the disease in children can be explained by characteristics of the innate response and the adaptive immune response, a low level of concomitant diseases, and, to a lesser degree, presence of cross-reactive T cells, caused by previous coronavirus infections (HCoV-229E, -OC43, -NL63, and -HKU1), which were as a viral infection [36]. However, there are cases of severe infection, which are associated with concomitant diseases as in adults [37].

Since April 2020, reports started to appear about the development of multisystem inflammatory syndrome in children (MIS-C) associated with COVID-19. MIS-C is a post-infection febrile pediatric hyperinflammatory condition [36]. According to a report issued by the Centers for Disease Control and Prevention in 2020, there were more than 2600 cases MIS-C. By the end of June 2022, in just the USA, there were 8639 cases of this syndrome and 70 deaths [38,39]. An interesting fact was revealed in the analysis of MIS-C: that it developed in asymptomatic children as well as those with severe COVID-19 [40,41]. Factors associated with the development of MIS-C after infection of SARS-CoV-2 have not been studied yet. The role of concomitant diseases in MIS-C development is not even clear [42], despite some authors’ statements that overweight patients can have a higher chance of MIS-C development [31,39]. The results from a number of studies have shown that patients were healthy previously and only rarely have chronic diseases such as bronchial asthma or autoimmune disease [43,44,45,46,47].

It should be noted that in some case series, there is a high proportion of African American patients and Latin American as well [42]. According to some data, MIS-C develops in genetically predisposed individuals due to a hyperinflammatory reaction after COVID-19 and affects several organs, including the cardiovascular system [27]. It was observed hypotension and shock (20–100%) in some patients or as a result of severe myocardial dysfunction or as a result of systemic hyperinflammation or vasodilation. The expansions of the coronary arteries or aneurysm were described in 6–24% of cases and arrhythmia in 7–60% [42]. Systolic left ventricular dysfunction (LVSD) was described in the majority of children with a MIS-C diagnosis. For example, in the first case series of MIS-C registered in the UK, there was cardiac dysfunction in 6 out of 8 patients (75%) [44]. In the later series, ventricular dysfunction was registered in 35–100% cases in children with MIS-C [32,42].

## 4. Pathogenesis of Myocardial Damage against the Background of COVID-19

Mechanisms of myocardial dysfunction development in MIS-C are not fully understood. Some scientists consider that the reason for damage in the myocardium in adults is hypoxic and ischemic damage, caused by damage to the coronary microvessels or coronary artery disease, acute myocarditis, or the syndrome of a systemic inflammatory reaction. Additionally, congenital heart disease can influence the course of COVID-19 [43].

From the viewpoint of pathophysiology, probably, an acute infection leads to acute cardiac injury and after that to the development of a post-viral immunological reaction and systemic hyperinflammation which leads to the emergence of inflammation and dysfunction of the myocardium in predisposed individuals [40]. Dilatation of a coronary artery which is detected in some part of patients in the acute phase of the illness may be associated with vasodilation in coronary arteries against the background of fever and inflammation. In addition, some patients have larger and giant aneurysms of coronary arteries. This necessarily involves the constant monitoring of these patients in relation to risk of an intimate tear of a coronary artery. Rhythm disturbance is registered in 7–60% patients with MIS-C. It has also been reported about hemodynamically significant arrhythmias [42,44].

There was myocardial damage in the range of 13–41% in hospitalized adult patients with COVID-19 which causes high mortality from cardiovascular complications [48]. However, current data on the tropism of SARS-CoV-2 to the myocardium is different. Thus, the study of pathomorphological material revealed an increased number of macrophages without lymphocytic myocarditis or significant myocyte necrosis, which is typical for other cardiotropic viral infections, including enteroviruses [49,50]. Single descriptions of myocarditis in cases of death in adults have demonstrated the presence of not only lymphocytic infiltration and antiviral antibodies, but also viral RNA in cardiac tissue. It is likely that there is a combination of direct viral damage to cardiomyocytes and hyperinflammation and cytokine storm of mediated myocardial dysfunction, combined with microthrombosis and vasculopathy [51,52,53].

According to some studies, it has been demonstrated that the main morphological manifestation of myocardial damage in COVID-19 is the so-called endotheliitis with dysplasia and activation of endotheliocytes, leading to hemorrhages, thrombosis of intramural arteries and necrosis. At the same time, no significant association was found between inflammatory markers and morphologically by confirmed myocarditis, and no convincing evidence was obtained on the direct involvement of the SARS-CoV-2 virus in the induction of myocarditis. This fact once again confirms the theory of the pathogenesis of a severe course of COVID-19, based on predominantly immune damage to the vascular endothelium and the formation of microthrombosis [48].

Besides this, endoteliitis contributes to narrowing of small vessels due to decrease of vasodilators’ production. It also stimulates micro- and macroembolization of lungs in combination with coagulopathy. It may lead to thrombosis of lung vessels, including small vessels, and become the reason of acute lung hypertension, while lung hypertension with simultaneous right heart ventriculus’ disfunction or without it is associated with mortality increase [49].

It is difficult to analyze the frequency of myocardial damage in children in various variants of the course COVID-19 that is primarily attributed to hospitalization of the majority of patients in emergency departments. According to one theory, getting the virus into the host cell is made easier by binding of the spike protein with ACE2 receptor [54]. The presence of virus causes a local inflammatory reaction with T infiltration and B lymphocytes [55]. In the early clinical phase of the infectious replication process of the virus in the cells of the myocardium causes, cellular damage and exposure to latent antigens in the systemic circulation—cardiac myosin that can lead to the autoimmune response [54]. Regardless of the condition of a patient, some of them can have the second phase, related to the exacerbation of inflammation in the heart tissue even if the viral infection was completely eliminated—post infectious myocarditis. Persistent cell damage can lead to the formation of fibrosis and finally, it can progress from dilated cardiomyopathy [52,55]. It is expected that hyperproduction of interferons and the enhanced cytokine response of T cells 1 and 2 types contributes dysfunction of the myocardium. In addition, the researchers found that key receptors ACE2 for the pathogenesis are on the surface of macrophages, and they indicated interaction of SARS-CoV-2 with macrophages CD68+, which shows the direct viral infection of these cells [50,54].

## 5. Multisystem Inflammatory Syndrome Associated with COVID-19 in Children: Myocardial Damage

MIS-C in children, associated with COVID-19 is a new syndrome in practice of pediatricians all around the world. It is registered in children within 2–6 weeks of the virus infection SARS-CoV-2 [56,57]. By October 2022, according to The Centre for Disease Control and Prevention (CDC), the number of children with MIS-C tripled in comparison with 2021 data where 74 cases were lethal because of the complications. Moreover, the average age of children was 9. In half of episodes there were children at the age of 5–13 years old, and 60% of cases were in boys [58,59].

MIS-C is associated with expressed immune activation and many potential mechanisms of immunopathogenesis and as it was mentioned above, the most commonly associated with damage of the gastrointestinal tract, skin, mucous membranes, and the cardiovascular system [60,61].

Signs of MIS-C are variative and include fever in 100% of cases; rush in 45–76% that is as a rule stable; different symptoms of affecting the gastro-intestinal tract (vomiting, diarrhea, abdominal pain) in 60–100%; conjunctivitis in 30–81% of cases; involvement of mucous (red or swollen lips, strawberry tongue) in 27–76% cases; neurocognitive symptoms (headache, apathy, confusion) in 29–58% cases; respiratory symptoms in 21–65% cases; sore throat in 10–16%; myalgia in 8–17%; swollen arms or feet in 9–16%, and lymphadenopathy in 6–17% cases [62].

According to some studies, there is some degree of heart involvement in children with MIS-C that is characterized by different amounts of increase in markers of myocardial damage, anomalies by the result of ECG, Echocardiography (Echo), and MRI [31,40].

Currently, long-term cardiovascular effects of MIS-C are not known yet. Patients have both acute and subacute complications and cardiovascular manifestations vary from mild to severe [63]. Thus, the results of a systematic review with meta-analysis, where there were 27 studies and it included 917 patients with MIS-C, the average age was 9.3 years old (95% confidence interval (CI), 8.4–10.1) [64]. There were symptoms of heart failure: dysfunction of the myocardium in 55.3% of cases (95% CI, 42.4–68.2), coronary artery aneurysms in 21.7% of cases (95% CI, 12.8–30.1) and shock in 65.8% of cases (95% CI, 51.1–80.4), accompanied by a significant increase in inflammatory and cardiac markers [64]. In addition, in 7–60% of cases patients had a heart rhythm disorder, changes in the ST segment, prolongation of the QT interval, and atrial and ventricular extrasystoles when MIS-C [37].

In laboratory tests, there has been a significant increase in C-reactive protein, Procalcitonin, ferritin, erythrocyte sedimentation rate, interleukin 6 (IL-6), and fibrinogen. There was a notable increase in the level of B-type natriuretic peptide, NT-proBNP and troponin in cardiac markers. In addition, the majority of patients had an increase in D-dimer level, neutrophils, a decrease in lymphocytes, and low albumin levels [64].

It should be noted that MIS-C can look like Kawasaki disease, based on clinical and laboratory signs. However, the high frequency of damage is typical for MIS-C in African Americans and Latin Americans and the prevalence in older age (the average age is 9) and, probably, a higher incidence of myocarditis, an elevation of troponin, ventricular dysfunction, and need for vasopressor drugs [63].

According to some data, in 50–70% cases in patients with MIS-C, there is myocarditis [39]. Son et al., have analyzed 518 children with MIS-C in their study and showed that nearly half of them needed in vasopressor support, in 42% of cases LV dysfunction was revealed, in 13% of cases, coronary artery aneurysms developed [65]. A treatment with immune globulins in combination with glucocorticoids was associated with a lower risk of new or persistent cardiovascular dysfunction than with only immunoglobulins therapy. When describing a case series—20 patients with MIS-C with cardiac failure—half of them had a decrease in the systolic LV function less than 55% according to Echo and myocardial edema, which demonstrates myocarditis, according to MRI [66].

A number of studies have shown that myocarditis, associated with MIS-C, proceeds more easily than myocarditis in adults against the background of SARS-CoV-2 [67]. Patients had a mild increase of troponin and then a rapid restoration of the LV function against the therapy background [27,37,64,68]. Nevertheless, there is evidence that diastolic dysfunction can last for 6 months after an acute disease in some patients [63].

It is assumed that in acute COVID-19, an increase of cardiac troponin levels can be the result of direct myocardial cell damage by the virus or consequences of the cytokine storm caused inflammatory responses that leads to myocarditis [64]. Chen et al. showed a sharp increase in the level of IL-6 in patients with COVID-19 with myocarditis [69]. IL-6 plays a crucial role in the cytokine storm, linked with COVID-19, forming hyper-inflammation and leading to the production of plasminogen activator inhibitor-1 (PAI-1), activator of the blood coagulation cascade. Moreover, it was demonstrated that inhibiting the transduction of IL-6 signals in a therapy by tocilizumab decreased the production of plasminogen activator inhibitor-1 and resolves clinical manifestations in acute form of COVID-19. Therefore, the potential mechanism of myocardial damage in MIS-C can be the cytokine storm, induced IL-6 that leads to fulminant myocarditis development [64,70]. However, it should be noted that the cytokine storm in addition to worsening of COVID clinical course leads to T-cell activation which release cytokines for maintaining strengthening of immune response [68].

Whereas development of MIS-C in adults is associated with poor prognosis [71], data on outcome prognosis and long-term outcome forecast in children with MIS-C is not widely known. Data of some research shows almost full recovery of heart function in the majority of children with MIS-C-associated cardiac involvement [31,32,33]. Taking into account the impact of facilitated immune response and the “cytokine storm”, intravenous immunoglobulins, hormones, and inotrope drugs are used for therapy acute decompensated of heart function. When is needed, artificial lung ventilation and extracorporal membrane oxygenation are used [31,32].

## 6. Features of the Immune Response in Myocardial Damage by SARS-CoV-2 

It is known that various immunocompetent cells, including both resident inflammatory cells of peripheral tissues (for example macrophages, dendritic cells, resident memory T-lymphocytes and so on) and also circulating in the peripheral blood immune cells, play an important role in the immunopathogenesis of myocarditis [71,72]. A huge number of different immune cells recruits from peripheral blood during the acute phase of a viral infection into infected myocardial tissue, which include monocytes and dendritic cells as well as various subpopulations of B- and T-lymphocytes [73]. Successful elimination of the virus closely related to mass destruction of virus-infected myocardial cells by effector cells of the immune system may contribute to the development of acute myocarditis, while the survival of virus-infected cells can lead to a persistent infection that can be a reason for the formation of chronic myocarditis, respectively [74].

Moreover, in the case of acute myocarditis in tissues of myocardium, there is the proinflammatory microenvironment, contributing to the effective elimination of the pathogen, and with successful destruction of it, the processes of repair and regeneration of inflamed tissues are launched. These processes closely related to attraction to the inflamed myocardium from circulating immune cells of different subpopulations. The balance between them determines not only the effectiveness of the course of protective reactions, but also the launch of the reparative processes.

Traditionally, the key role in the destruction of intracellular pathogens with virus involves various effector cells (T-helper type 1 (Th1), cytotoxic CD8+ T-lymphocytes, natural killer cells) involved in the implementation of inflammatory reactions of type 1 [75,76]. Th1 participates in the implementation of cellular reactions of acquired immunity through the production of pro-inflammatory cytokines IFNγ and TNFα and also IL-2 and IL-15, while the main target cells are tissue macrophages which acquire the M1 phenotype, and cytotoxic cells—CD8+ T-lymphocytes and NK-cells [77]. The role of Th1 cells in pathogenesis of COVID-19 is quite contradictory. For example, some authors point to the positive role of IFNγ-producing Th1 cells with this pathology and associate their increased activity with a milder course of the disease [78]. On the other hand, in adult group of patients, which is traditionally characterized by a severe course of COVID-19, there was a decrease in the levels of IFNγ-producing virus-specific cells. It also indirectly indicates the important role of Th1 cells in the development of an effective immune response [79].

The importance of Th1 cells has also been noted in the case of experimental myocarditis in the formation of this pathology. When it was shown that cells are unable to respond to IL-12 by forming Th1 cells from “naive” Th0 (deprived of signal due to blockade of IL12Rβ1 and STAT4), myocarditis did not develop under experimental conditions in mice [80]. However, it was shown in the framework of the study already mentioned that blockade of key effector cytokine Th1 cells—IFNγ—due to knock-out or monoclonal antibodies in vivo contributed to the progression of myocarditis in mice.

The experiment showed that in mice with IFNγ deficiency, there is a development of inflammatory dilated cardiomyopathy accompanied by severe fibrosis and even the development of constrictive hemodynamic complications. IFNγ protected against the development of severe chronic myocarditis, pericarditis, and dilated cardiomyopathy after infection with Coxsackievirus B3, reducing mast cell degranulation, volume of fibrous myocardial tissue and production of profibrotic cytokines such as TGFb, IL-1b and IL-4 [81]. Thus, the role of Th1 in the pathogenesis of myocarditis remains highly controversial.

In further studies of experimental myocarditis, it was found that mice with double deficient IL-23 and IL-12 (IL12p40 -/-) were protected from the development of experimental autoimmune myocarditis in contrast to mice with deficiency only in IL-12 (IL12p35 -/-). This implies direct participation of IL-23 in the pathogenesis of myocarditis [75,82]. IL-23, together with IL-1β, IL-6 and TGFb play the important role in “polarization” of Th0 to the side Th17 and the development of type 3 inflammation [77]. Type 3 cellular immune response (mediated by Th17 and neutrophils), aimed at eliminating extracellular bacteria and fungi, is characterized by an influx of neutrophils from the peripheral blood into the inflamed tissue, as well as the activation of barrier tissue cells (primarily mucosal epithelial cells) with increased mucus production and antimicrobial protective factors [77]. It should be noted that the level of these key polarizing cytokines (IL-1β and IL-6) especially increases in the acute phase of the infectious process, caused SARS-CoV-2, that can serve as additional markers of the severity of the disease [83,84,85].

The main effector cytokines of Th17 are proteins of the IL-17 family (primarily IL-17A), regulating functions of neutrophils and their attraction to the focus of inflammation, and IL-22, the main function of which is the activation of the protective functions of the cells of the epithelial layers, moreover, precisely IL-17A [86] and IL-22 [87] may play an important role in the pathogenesis of COVID-19 and be considered as targets for the treatment of this disease.

It was shown that in CD4+ peripheral blood T-lymphocytes of patients with severe COVID-19, there was decreased expression of Th17-associated genes on the example of RORC, IL17A, IL17F and CCR6 [88]. The minimum level of Th17 was observed in patients with severe COVID-19, moreover, within the framework of the general pool of CCR6+ Th17, it was exactly noted in severe patients that there was a decrease in the proportion of CCR4− CXCR3+ Th17.1 cells and an increase in CCR4+ CXCR3—“classical” Th17 [89].

In this way, Th17 can migrate to inflamed tissues where after pathogen recognition, they produce effector cytokines and promote the migration of neutrophils into lung tissue [90,91]. In the case of viral myocarditis, it has been shown that the role of IL-17A as a pro-inflammatory Th17 mediator is not key in the development of acute inflammatory stages of myocarditis, at least in mice [92]. On the other hand, in this study, it was found that IL-17A is required for the progression of acute myocarditis in dilated cardiomyopathy, and contributes to the development of foci of fibrosis in the myocardium and loss of cardiac function. Moreover, it was shown that another important effector cytokine Th17—granulocyte-monocyte colony-stimulating factor (GM-CSF)—in combination with IL-17A—increases infiltration of the inflamed myocardium by monocytes in the model of experimental autoimmune myocarditis [93]. Similar results were obtained in studies of patients with myocarditis and its long-term complications in the form of dilated cardiomyopathy [94]. Thus, in the peripheral blood of patients, there was an increase in the level of CD4+ IL17+ T-cells, CD4+ T-cells, and producing GM-CSF as well as an increase in blood serum IL-6, TGF-β and IL-23—key cytokines, responsible for the polarization of Th17. In addition, persistent heart failure was strongly associated with high levels of circulating Th17 and their “polarizing” cytokines, as well as a decrease in the level of regulatory T-lymphocytes which could also contribute to an increase in the clinical manifestations and severity of the disease [95].

Cellular immune response type 2 (Th2 mediated), characterized by the flow of eosinophils into the inflamed tissue, mast cells, basophils and alternatively activated macrophages (M2), as well as remodeling of mucosal tissues with an increase in the proportion of mucus-producing cells, increased the contractility of smooth muscle cells and eventually the development of fibrosis [77].

In the case of experimental animals, which had no responses, Th1 mediated and Th17 (IL17A^−/−^ IFNγ^−/−^), virus-initiated development of myocarditis was accompanied by activation of Th2 cells and the development of eosinophilic myocarditis, clinically similar to human myocarditis [95]. The main cells that caused the destruction of cardiomyocytes and damage to the extracellular matrix were activated effector cytokines of Th2 eosinophil cells. Thus, the blockade of IL-4, one of the key effector cytokines of Th2 in mice with experimental autoimmune myocarditis, accompanied by a decrease in the severity of the disease that was expressed in a decrease in the proportion of Th2 and an increase in the proportion of Th1 cells, reduction in vitro production IL-4, IL-5 and IL-13 against the background of increased synthesis and secretion of IFNγ [96]. On the other hand, IL-13 had expressed protective effects in the induction of myocarditis, caused either by immunization with cardiac myosin peptide, or CB3 virus infection in BALB/c mice [97]. Severe myocarditis occurring in the absence of IL-13, characterized by increased leukocyte infiltration of myocardial tissues, increased levels of pro-inflammatory IL-1b and IL-18 (but not IL-17), increased levels of autoantibodies to cardiac myosin and severe cardiac fibrosis was considered as the development of severe dilated cardiomyopathy with impaired cardiac function in IL-13 knockout mice and was fatal in most cases.

In acute new coronavirus infection, an increase in the proportion of T-helpers was also noted in the peripheral blood of patients, expressing CCR4 on their membrane and GATA3 in the nucleus [98], and high levels of Th2 cytokines are detected in the blood serum [99]. An increase in the blood levels of Th2 cells with the CXCR3− CCR6− phenotype was closely associated with the severe course of the disease [83] and poor outcomes in patients with severe COVID-19 [100].

Moreover, in patients who recovered from COVID-19, high levels of Th2 cells persisted in the blood for several months, although the levels of IL-4, IL-5 and IL-13 were not significantly different from control values [101]. It can be assumed that the development of the type 2 inflammatory process associated with an increase in Th2 and eosinophils in peripheral blood can be considered as a favorable prognostic factor. Moreover, there is evidence that Th2 and eosinophils, through cytokine secretion (in the first turn, IL-13) are able to reduce the level of ACE2 expression on epithelial cells—key targets for the SARS-CoV-2 virus [102]. In a whole series of studies, the relationship between eosinophilia and a mild form of COVID-19 was noted that indicates the important role of these cells in limiting inflammation in this infectious process [103,104]. On the other hand, restriction or blockade of the activation of other important Th2 target cells—mast cells, associated with the secretion of inflammatory mediators and the production of pro-inflammatory cytokines and chemokines, can be used in clinical practice to reduce the volume of lung tissue damage [105].

## 7. Features of Heart Lesions Diagnosis in COVID-19

The main sign of heart damage is an increase in troponin levels I, or troponin T. An increase in cardiac markers may also indicate the heart damage as a result of severe hypoxemia or coagulopathy, leading to acute coronary syndrome and endothelial dysfunction with decreased production of nitric oxide [106]. In a systematic review of four studies, (374 patients), cardiac troponin I levels were significantly higher in patients with severe COVID-19 in comparison with patients with mild disease (OR 25.6, 95% CI, from 6.8 to 44.5) [49].

To diagnose a lesion of myocardium (by ECG and ECHO it cannot be argued that these are inflammatory changes), various non-invasive instrumental research methods where ECG and echocardiography are the most accessible [106,107]. It should be noted that according to the ECG data, it is possible to register sinus tachycardia, nonspecific changes in repolarization processes (ST segment elevation, inversion of the T wave), various arrhythmias and conduction disorders [107,108,109]. Cardiovascular disorders were examined in 53% of cases after COVID-19 and in 13%, showed persistent decrease in left ventricular ejection fraction (LVEF) after 6 months [107].

Echocardiography reveals regional wall motion disorders, dilatation of the chambers of the heart, and regional or diffuse dysfunction of the left and right ventricles. In addition, pericardial effusion of varying degrees can be detected. It was also found that the longitudinal deformation of the left ventricle correlates with subclinical myocardial dysfunction after 6 months that was detected by an MRI of the heart in the follow-up period in 33% of cases [107]. The elevation of myocardial necrosis markers and reduction of LVGLS and the presence of LGE on CMR was detected in quarter of MIS-C patients [109]. However, ECG and echocardiography are significantly less sensitive than MRI [108].

An MRI of the heart is another method for diagnosing myocarditis in patients with COVID-19 [68,106]. In 2020, the Society for Cardiovascular Magnetic Resonance Imaging proposed a protocol for assessing myocarditis with infection of COVID-19 both in children and adults [110]. MRI results in cases of acute myocarditis associated with COVID-19 do not differ from what is described in the LLC criteria [107,111]. In addition to identifying structural anomalies, an MRI can assess the presence of edema and myocardial necrosis as well as late enhancement with gadolinium which are pathognomonic signs. It should be noted that changes in MRI may persist during the convalescent period [67,110].

In addition to MRI for detailed diagnosis of cardiac muscle involvement and coronary vessels in MIS-C, it is possible to perform multi-slice computed tomography of the heart with angiography as well as myocardial scintigraphy [106]. In the pediatric population, taking into account the radiation exposure to the child’s body, the use of MRI with intravenous contrast and cardio synchronization is certainly the most appropriate.

In order to confirm the diagnosis and exclude coronary heart disease as one of the possible causes of elevated troponin levels it is possible to use a coronary angiography. Positron emission tomography (PET) is rarely used in clinical practice for the diagnosis of myocarditis and may reveal characteristic inflammation in the myocardium. Endomyocardial biopsy, although critical to the diagnosis, is rarely used in patients with COVID-19 especially, in the pediatric population due to the high risk of complications during the procedure [106,111]. However, histological examination reveals signs of interstitial and endocardial inflammation. Ultrastructural examination reveals single or small groups of viral particles in structurally damaged interstitial cells. Additionally, large vacuolated CD68+ macrophages are detected [111].

Against the background of a large arsenal of methods for diagnosing myocardial damage, there are major difficulties because of the lack of data on the most effective methods and criteria for diagnosing myocardial injury in the pediatric population during and after COVID-19 [55,112].

Despite unprecedented collective efforts, the issues of choosing effective and sensitive diagnostic methods are still very relevant and require further research.

## 8. Post-COVID-19 Syndrome or Long-COVID-19: Heart Disease in Children

Among the long-term consequences of SARS-CoV-2 infection in the pediatric population two are of greatest concern: MIS-C and Long COVID or Post-COVID-19 syndrome [113,114]. Long-COVID-19 or Post-COVID-19 syndrome refers to the development of persistent symptoms after COVID-19, described more often in adults, which involve the development of cognitive, endocrine and cardiorespiratory symptoms [113]. To date, there is no clear definition of this syndrome and there is no consensus on the duration of symptoms justifying the diagnosis that ranges from 4 to 12 weeks after acute infection [115]. It should be noted that Post-COVID-19 usually presents with a group of symptoms that overlap and change over time and can affect any body system (https://app.magicapp.org/#/guideline/EQpzKn/section/n3vwoL, accessed on 5 October 2022).

Definition of Long-COVID-19 or Post-COVID-19 in children and youth based on the Delphi Consensus, but according to the WHO definition, was shown in the study of Stephenson et al. [116].

Data on the prevalence of Long-COVID-19 in the pediatric population is scarce and inconsistent, ranging from 4% to 66%. In this regard, it is quite difficult to predict children who will have the development of Long-COVID-19 [117]. In addition, Long-COVID-19 studies are mostly based on symptoms which are reported by parents, including those without previously confirmed SARS-CoV-2 infection [117,118]. One of the first reports of Long-COVID-19 in children was a case of five children from Sweden aged 9 to 15 where symptoms continued for 6 to 8 months after acute infection. None of the patients required hospitalization in acute phase, but one girl with comorbidities was hospitalized after an acute infection due to perimyocarditis [119].

Among 320,825 people with Long-COVID-19 in the UK, 0.16% of cases are in children aged 2–11; 0.65% in children aged 2–16; and 1.22% of cases are in children aged 17–24. A large national study of Long-COVID-19-in children CLoCk 1 showed that 3 months after testing for COVID-19, 66.5% of children and youth who had a positive test and 53.3% of children and youth who had a negative test were still symptomatic while 30.3% and 16.2%, respectively, had three or more symptoms [116].

In their work, Zimmermann et al. analyzed 14 studies which examined the symptoms of Long-COVID-19 in 19,426 children and adolescents. The most common symptoms were headache (3–80%), fatigue (3–87%), sleep disturbance (2–63%), difficulty concentrating (2–81%), abdominal pain (1–76%), myalgia or arthralgia (1–61%), nasal congestion or runny nose (1–12%), cough (1–30%), chest tightness or pain (1–31%), loss of appetite or weight (2–50%), impaired sense of smell or anosmia (3–26%), and rash (2–52%) [94,95].

Thus, in addition to heart signs at Long-COVID-19, neurological symptoms (emotional lability, etc.), psychiatric complications (depression, anxiety, etc.), respiratory complications (chest pain, cough, etc.), ENT-system (anosmia, etc.), and gastrointestinal tract (vomiting, diarrhea, abdominal pain, etc.) are described [120]. Zimmermann and Fainardi, in studying the risk factors for Long-COVID-19 in children according to the literature (14 studies), found a positive correlation between increasing age and symptoms of Long-COVID-19 as well as female gender, the presence of allergies, deterioration in physical and mental health before infection, and the prevalence of persistent symptoms. It should be noted that in most studies, symptoms persisted for no longer than 12 weeks, with an average of 8 weeks before most symptoms disappeared [112,113].

The main mechanisms that are responsible for the varied clinical presentation of Long-COVID-19 are still unknown [109], although such disturbances are observed even in asymptomatic patients during the acute phase of the disease [119].

As pathogenetic mechanisms, dysregulation of the immune system with a hyperinflammatory state, direct viral toxicity, damage to the endothelium and microvessels were considered [121]. However, an altered immunological response appears to play a leading role.

For example, Th17 hyperactivation and disturbances in their subpopulation composition, changes in the ratio of “regulatory” and “pro-inflammatory” Tfh cells as well as a decrease in the control of antibody-producing B cells are very similar to changes, characteristic of a wide range of autoimmune pathologies [122,123], the incidence of which increases sharply after COVID-19 [124]. Long-term disturbances in the processes of maturation and differentiation of NK-cells and cytotoxic T-lymphocytes, the presence of inhibitory receptors or markers of “cellular aging” on their surface which is accompanied, first of all, by low efficiency of destruction of target cells may reduce the effectiveness of antitumor and antiviral immunity [125,126,127]. In addition, hyperactivation of tissue macrophages, the formation of a pool of activated monocytes migrating from the bloodstream against the background of a cytokine “storm” and a change in the balance between T-helpers of different populations (Th1/Th2 and Th17/Treg) in the focus of inflammation contribute to the disruption of the regeneration of inflamed tissue of different localization and development of fibrosis [128,129].

In one of the recent study immunological differences were described between children who completely recovered from acute infection, and children with Post-COVID-19 (PASC). For example, when comparing children with PASC and convalescents, distinctive immunological features were revealed. The PASC group showed significantly higher levels of plasmablasts, IgD-CD27+ memory cells, and switched IgM-IgD-B cells. In recovered children, significantly more naive and unswitched subpopulations of IgM+ IgD+ and IgM+ CD27-CD38dim B cells were identified. The T-regulatory compartment showed no significant difference. In addition, serum levels of IL6 and IL1β were elevated in patients with PASC and consistently higher than in children who recovered from infection [130].

Nowadays, in controlled or large cohort studies, no superiority between treatment agents in therapy of Long-COVID-19 is demonstrated. Therapy, as a rule, is symptomatic [131]. Despite literature data showing that the prognosis in children is favorable, not much time passed yet from the onset of the pandemic to make reliable conclusions on long-term outcomes of Long-COVID-19 in children [109,110].

Today, Long-COVID-19 in the pediatric population is a significant and real problem. Without doubt all children with suspected or documented COVID-19 infection and their parents, even in the absence of symptoms at the time of diagnosis, should be informed about the possible persistence of symptoms for more than 4 weeks and their possible reappearance after the acute phase. It should be remembered that girls and adolescents with comorbidities, including psychiatric conditions, are associated with a higher risk of prolonged COVID-19 [110]. However, current knowledge on the pathogenesis, diagnosis, treatment, and prevention of Long-COVID-19 in children is lacking. The presented high heterogeneity of clinical symptoms and involvement of various organs and systems, including cardiovascular, in Long-COVID-19 in the pediatric population requires targeted research to better study and understand these issues.

## 9. Conclusions

Outcome of COVID-19 pandemic still requires investigation. Currently, analysis of long-term consequences of SARS-CoV-2’s impact on the body of children and adults is ongoing. Approximately 20–55% of patients in young and middle age with COVID-19 were hospitalized, and up to 18.5% of them had a severe course of the disease [132], wherein patients with cordial and vessels pathology, especially children, including children with congenital pathology, take special place [43,132].

**Figure 1:**
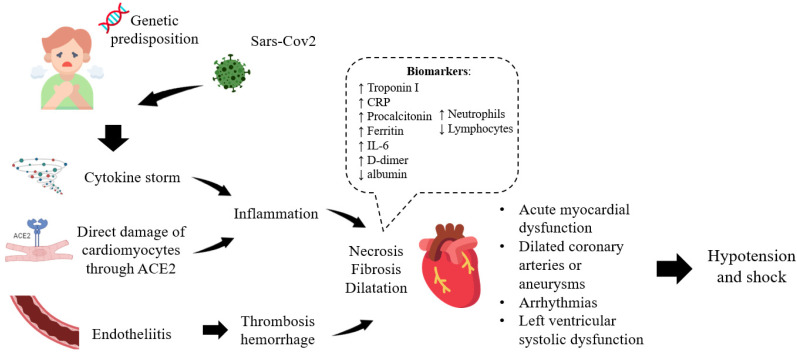
Mechanism of heart damage in COVID-19.

Thus, extensive evidence suggests that the heart damage in COVID-19 is triggered by systemic hyperinflammation caused by viral infection (Figure 1).

It should be noted that understanding of the immune response, diagnosis and treatment of COVID-19 is highly oriented towards the adult population. At the same time, despite the fact that COVID-19 in children usually occurs in a mild form, there is an incomplete understanding of the course as an acute infection and its subsequent manifestations such as Long-COVID-19 or Post-COVID-19 (PASC) in the pediatric population, correlations with comorbidities and immunological changes. It should be added that the clinical manifestations and mechanisms of damage, including the heart, both against the background and after COVID-19 in children differ from those in adults.

Further studies are needed to evaluate whether transient or persistent cardiac complications are associated with long-term adverse cardiac events. In addition, at the moment, the question of the most effective methods for diagnosing myocardial damage in the pediatric population during and after COVID-19 is open.

## Data Availability

Not applicable.
